# Methodological Issue of Mitochondrial Isolation in Acute-Injury Rat Model: Asphyxia Cardiac Arrest and Resuscitation

**DOI:** 10.3389/fmed.2021.666735

**Published:** 2021-04-12

**Authors:** Tomoaki Aoki, Yu Okuma, Lance B. Becker, Kei Hayashida, Koichiro Shinozaki

**Affiliations:** ^1^The Feinstein Institutes for Medical Research at Northwell, Manhasset, NY, United States; ^2^Department of Neurological Surgery at Fukuyama City Hospital, Fukuyama, Japan; ^3^Department of Emergency Medicine, Donald and Barbara Zucker School of Medicine at Hofstra/Northwell, Hempstead, NY, United States

**Keywords:** mitochondria, mitochondrial dysfunction, mitochondrial isolation, oxygen consumption, ischemic reperfusion injury

## Abstract

**Background:** Identification of the mechanisms underlying mitochondrial dysfunction is key to understanding the pathophysiology of acute injuries such as cardiac arrest (CA); however, effective methods for measurement of mitochondrial function associated with mitochondrial isolation have been debated for a long time. This study aimed to evaluate the dysregulation of mitochondrial respiratory function after CA while testing the sampling bias that might be induced by the mitochondrial isolation method.

**Materials and Methods:** Adult rats were subjected to 10-min asphyxia-induced CA. 30 min after resuscitation, the brain and kidney mitochondria from animals in sham and CA groups were isolated (*n* = 8, each). The mitochondrial quantity, expressed as protein concentration (isolation yields), was determined, and the oxygen consumption rates were measured. ADP-dependent (state-3) and ADP-limited (state-4) respiration activities were compared between the groups. Mitochondrial quantity was evaluated based on citrate synthase (CS) activity and cytochrome c concentration, measured independent of the isolation yields.

**Results:** The state-3 respiration activity and isolation yield in the CA group were significantly lower than those in the sham group (brain, *p* < 0.01; kidney, *p* < 0.001). The CS activity was significantly lower in the CA group as compared to that in the sham group (brain, *p* < 0.01; kidney, *p* < 0.01). Cytochrome c levels in the CA group showed a similar trend (brain, *p* = 0.08; kidney, *p* = 0.25).

**Conclusions:** CA decreased mitochondrial respiration activity and the quantity of mitochondria isolated from the tissues. Owing to the nature of fragmented or damaged mitochondrial membranes caused by acute injury, there is a potential loss of disrupted mitochondria. Thus, it is plausible that the mitochondrial function in the acute-injury model may be underestimated as this loss is not considered.

## Introduction

Cardiac arrest (CA) is a major public health issue affecting approximately 600,000 patients each year in the United States (1). Various pathophysiological changes occur during and after CA, including mitochondrial dysfunction, multiple organ failure, and prolonged neurological dysfunction, that increase the mortality of patients with CA (2). Studies have suggested that therapies effective against mitochondrial dysfunction may improve mortality and neurological damage after CA in rodent models (3, 4). These results implicate the mitochondria as effectors or targets to improve the survival of patients with CA. Thus, studies focusing on mitochondrial pathophysiology are imperative to understanding the mechanisms underlying complex biological responses preceded by ischemia/reperfusion injury.

Mitochondrial research using experimental animal models plays an important role in the study of mitochondria, and mitochondrial isolation is the foremost method for studying the pathophysiology of mitochondrial dysfunction (5, 6). Therefore, the development of methods for mitochondrial purity assessment are currently in high demand, and different mitochondrial isolation methods have been reported by several investigators (7, 8).

Picard et al. (5) investigated the potential complications arising from isolation methods that involve structural and functional disruption of the mitochondria. They attributed the functional alterations induced by mitochondrial isolation to disruption of the mitochondrial morphology caused by mechanical homogenization as well as loss of soluble proteins and other molecules from the mitochondrial matrix. This is significant under experimental conditions given that animal models with acute injuries likely have mitochondrial disruption in nature. However, although Picard et al. discussed a potential risk of sampling bias as a result of the loss of disrupted mitochondria during the isolation process, no studies have shown such a bias in acute-injury animal models.

We previously reported responses to resuscitation of tissues, including the brain and kidneys, following prolonged CA by examining mitochondrial respiration using isolated mitochondria. The present study aimed to evaluate mitochondrial dysfunction in the brain and kidney tissues after CA while testing the sampling bias induced by our mitochondrial isolation method, which was performed by measuring multiple indicators of the purity of mitochondrial samples.

## Materials and Methods

The Institutional Animal Care and Use Committee of the Feinstein Institutes for Medical Research approved this study protocol. All methods were performed in accordance with the Guide for the Care and Use of Laboratory Animals, American Veterinary Medical Association Guidelines on Euthanasia and all other related regulations. This report is in compliance with the Animal Research Reporting of *In Vivo* Experiments guidelines.

### Generation of Rat CA Model and Interventions

All instrumentation and surgical preparations in this non-randomized, prospective, and experimental controlled study were performed according to our previously described protocols (9). In brief, 16 adult male Sprague–Dawley rats (450–550 g, Charles River Laboratories, MA,USA) were anesthetized with 4% isoflurane (Isosthesia, Butler-Schein AHS, OH, USA) and intubated with a 14-gauge plastic catheter (Surflo, Terumo Medical Corporation, NJ, USA). We used male rats to avoid potential hormonal or genetic differences among animal subjects and ensure that the observed differences could be attributed to the experimental intervention (i.e., to minimize potential sources of variability). Inhaled anesthesia was induced to rats and they were mechanically ventilated (Ventilator Model 683, Harvard Apparatus, MA, USA), and anesthesia was maintained with 2% isoflurane and a fraction of inspired O_2_ (FIO_2_) equivalent to 0.3. Core temperature was maintained at 36.5 ± 1.0°C during the surgical procedure. Animals were assigned to two groups: CA and sham (*n* = 8 for each group). The CA group included rats successfully resuscitated with cardiopulmonary resuscitation (CPR) after a 10-min asphyxia. The sham group included rats that were not treated with asphyxia or CPR. After cannulation through the left femoral vein, neuromuscular blockade was achieved by slow intravenous administration of 2 mg/kg vecuronium bromide (Hospira, IL, USA) for the CA group rats, and asphyxia was induced by turning off the ventilator. After 10 min, the animals were resuscitated by restarting mechanical ventilation at an FIO_2_ of 1.0 and performing manual chest compression CPR. Chest compressions were performed with two fingers over the sternum at a rate of 260–300 beats/min. Immediately after beginning CPR, a 20 μg/kg bolus of epinephrine was administered to the rats through the venous catheter. Following restoration of spontaneous circulation, defined as systolic blood pressure > 60 mmHg, CPR was discontinued. The same surgical procedures were performed for rats in the sham group, including vecuronium and epinephrine injections. Mechanical ventilation was discontinued 30 min post CPR; thereafter, the rats were euthanized, and tissues were collected for mitochondrial experiments.

All surgical procedures, including resuscitation, were performed by one investigator; therefore, blinding procedures were not performed. Allocation concealment is not possible when using an acute-injury model as opposed to healthy control animals. Therefore, we used sham-surgery animals as our control group to reduce the risk of exaggerated effects. The other investigator independently and unbiasedly performed the subsequent biochemical assays.

### Isolation of the Brain and Kidney Mitochondria and Evaluation of Mitochondrial Respiratory Function

Mitochondrial samples from the brain and kidneys were isolated from the sham and CA groups according to a modified procedure described by Scholte et al. (10). All the procedures were performed at 4°C. Briefly, excised tissues were immediately placed in mitochondrial isolation buffer composed of 210 mM mannitol, 70 mM sucrose, 10 mM HEPES (pH 7.3), and 0.2 mM EGTA with 0.2% w/v fatty acid-free bovine serum albumin (MESH-BSA). The spinal cord, extra ventricular tissue of the brain, and fats from both tissues were isolated in MESH-BSA buffer. Next, tissues were blot-dried on filter paper, weighed, and placed in MESH-BSA buffer. After mincing and washing, the tissues were diluted in MESH-BSA buffer, and subsequently homogenized using a Teflon motor-driven homogenizer (Model BDC2010, Caframo Lab Solutions, Ontario, Canada) at eight and three strokes for the brain and kidneys, respectively. Homogenates were centrifuged at 5,600*g* for 1 min, and supernatants were decanted into a polycarbonate tube and centrifuged again at 12,000*g* for 6 min. For brain samples, homogenization was performed twice, and the pooled supernatants were centrifuged. The brain tissue homogenization supernatants were gently decanted until the synaptosomes layer reached the top, and the remaining loose pellets were suspended with 20 mL of 12.5% Percoll (GE Healthcare, IL, USA) in MESH buffer without BSA and centrifuged at 12,000*g* for 6 min. Kidney homogenization did not require this process because of the lack of myelin synaptosome structures. For both the brain and kidney samples, supernatants were gently removed with pipettes without disturbing mitochondrial pellets (usually ~200 μL buffer remained). Finally, the pellets were resuspended in 20 mL MESH buffer and centrifuged at 12,000*g* for 6 min. After mitochondrial pellets were collected and their volumes measured, mitochondrial concentrations were determined using BCA assay with BSA as a protein standard, and then, isolation yields (mg protein/g tissue) were calculated.

Subsequently, the oxygen consumption was measured using a Strathkelvin oxygen electrode (30°C). Isolated mitochondria samples were diluted in an oxygen electrode mix buffer containing 80 mM KCl, 50 mM MOPS, 1 mM EGTA, 5 mM KH_2_PO_4_, and 1 mg defatted BSA/mL at pH 7.4 (11). ADP-dependent (state-3), ADP-limited (state-4), and DNP-dependent (uncoupled) respiration were measured in 150 μL mitochondrial suspension (0.5 mg/mL) using glutamate and malate as substrates. The rates of substrate oxidation were expressed as nmol/min/mg protein. The respiratory control ratio (RCR) was calculated as the ratio of state-3 to state-4 respiration.

### Citrate Synthase Activity Assay

The CS activity of each isolated mitochondrial sample was measured using a CS activity assay kit (MAK193, Sigma-Aldrich, MO, USA) according to the manufacturer's instructions. Briefly, after preparing the reagents, isolated mitochondrial samples were diluted with CS assay buffer. Diluted samples, reduced glutathione (GSH) standard solutions, and positive controls were added to 96-well plates, followed by reaction mixes containing CS developer and CS substrate mix. The specific absorbance at 412 nm was measured every 5 min for 30 min. Finally, CS activity was calculated according to the GSH amount, calculated using a standard curve, and the reaction time.

### Cytochrome C ELISA

The cytochrome c concentration in each isolated mitochondrial sample was measured using a cytochrome c profiling ELISA kit (ab110172, Abcam, Cambridge, UK), according to the manufacturer's instructions. Briefly, after preparing the reagents, isolated mitochondria samples were diluted with a solution containing 0.1% sodium dodecyl sulfate (SDS) and centrifuged at 15,000*g* for 20 min. Supernatants of samples and standards were added to the supplied 96-well microplates and incubated for 3 h. After the antigen–antibody reaction using a detector antibody against cytochrome c and HRP labels, HRP development solution was added. Absorbance was measured at 600 nm using a plate reader (Spark®, TECAN, Männedorf, Switzerland). Finally, the cytochrome c concentration in each sample was calculated according to a standard curve.

### Statistical Analysis

Data are shown as mean ± standard deviation (SD) for continuous variables. An unpaired two-tailed Student's *t*-test was used to compare two independent groups. Two-tailed ***p***-values were calculated, and statistical significance was set at *p* < 0.05. SPSS 25.0 (IBM, Armonk, NY, USA) was used to perform all statistical analyses.

## Results

### Cardiac Arrest Decreases Mitochondrial Respiratory Function

Tables 1, 2 show the basal characteristics and oxygen consumption rates, respectively, of isolated mitochondria from the brain and kidneys. Brain tissue weight of rats in the CA group was greater than that of rats in the sham group (2.03 ± 0.05 and 1.91 ± 0.06 g, respectively; *p* < 0.01), but kidney tissue weight did not differ between the two groups (1.63 ± 0.09 and 1.62 ± 0.09 g, respectively; *p* = 0.78). The isolated mitochondrial volume of the CA group was significantly lower than that of the sham group in the brain and kidney tissues (brain: 146 ± 42 and 196 ± 44 μL, *p* < 0.05; kidney: 554 ± 68 and 692 ± 113 μL, *p* < 0.05, respectively). Similarly, isolation yield of the CA group was significantly lower than that of the sham group in both tissues (brain: 2.71 ± 0.49 and 4.29 ± 0.82 mg protein/g tissue, *p* < 0.001; kidney: 18.4 ± 1.7 and 22.6 ± 1.3 mg protein/g tissue, *p* < 0.001, respectively).

**Table 1:** Basal characteristics and oxygen consumption rates of the isolated mitochondria from the brain.

**Brain**			
**Measurement**	**Sham (*****n*** **=** **8)**	**CA (*****n*** **=** **8)**	***p*****-value**
Rat weight (g ± SD)	509 ± 26	513 ± 18	0.7247
Tissue weight (g ± SD)	1.91 ± 0.06	2.03 ± 0.05	0.0010^**^
Mitochondria volume (μL ± SD)	196 ± 44	146 ± 42	0.0369^*^
Isolation yield(mg protein/g tissue ± SD)	4.29 ± 0.82	2.71 ± 0.49	0.0004^***^
State-3 activity(nmol/min/mg protein ± SD)	286 ± 50	209 ± 26	0.0016^**^
State-4 activity(nmol/min/mg protein ± SD)	45.4 ± 12.6	43.9 ± 14.7	0.8296
RCR ± SD	6.57 ± 1.42	5.17 ± 1.63	0.0881

*CA, cardiac arrest; SD, standard deviation; RCR, respiratory control ratio*.

* *p < 0.05 in all variables*.

** *p < 0.01 in all variables*.

*** *p < 0.001 in all variables*.

**Table 2:** Basal characteristics and oxygen consumption rates of the isolated mitochondria from the kidney.

**Kidney**			
**Measurement**	**Sham (*****n*** **=** **8)**	**CA (*****n*** **=** **8)**	***p*****-value**
Rat weight (g ± SD)	509 ± 26	513 ± 18	0.7247
Tissue weight (g ± SD)	1.62 ± 0.09	1.63 ± 0.09	0.7780
Mitochondria volume (μL ± SD)	692 ± 113	554 ± 68	0.0105^*^
Isolation yield (mg protein/g tissue ± SD)	22.6 ± 1.3	18.4 ± 1.7	< 0.0001^***^
State-3 activity (nmol/min/mg protein ± SD)	269 ± 55	148 ± 37	0.0001^***^
State-4 activity (nmol/min/mg protein ± SD)	42.8 ± 19.9	27.6 ± 6.5	0.0602
RCR ± SD	6.94 ± 1.96	5.75 ± 2.58	0.3166

*CA, cardiac arrest; SD, standard deviation; RCR, respiratory control ratio*.

* *p < 0.05 in all variables*.

*** *p < 0.001 in all variables*.

The state-3 respiration activities in the brain and kidney mitochondria of the CA group declined significantly compared to those in the sham group (brain: 209 ± 26 and 286 ± 50 nmol/min/mg protein, *p* < 0.01; kidney: 148 ± 37 and 269 ± 55 nmol/min/mg protein, *p* < 0.001, respectively). In contrast, we did not observe significant differences in state-4 respiration activities in either tissue. Thus, the RCR showed a decreasing trend after CA in both tissues.

### Cardiac Arrest Decreases Tissue Citrate Synthase Activity

**Figure 1:**
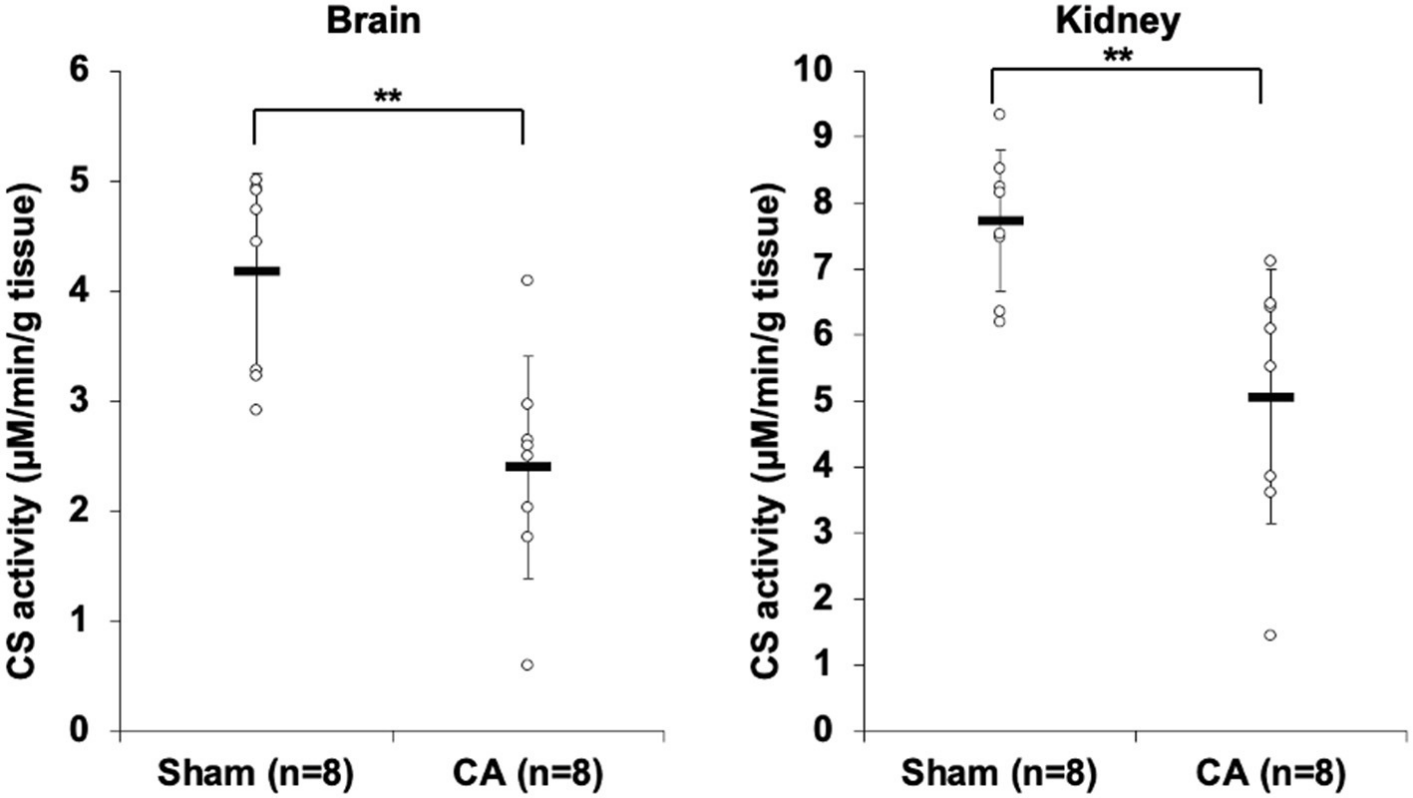
Results of citrate synthase activity assay comparing isolated mitochondria of cardiac arrest rats with that of sham rats. The CS activity of the brain and kidney mitochondria in the CA group declined significantly compared to that in the sham group (brain, 2.40 ± 1.01 and 4.19 ± 0.89 μmol/min/g tissue, *p* < 0.01; kidney, 5.07 ± 1.92 and 7.73 ± 1.07 μmol/min/g tissue, *p* < 0.01, respectively). **p < 0.01.

Figure 1 shows the results of CS activity assay of isolated mitochondria from the brain and the kidney. The CS activity of the brain and kidney mitochondria in the CA group declined significantly compared to that in the sham group (brain: 2.40 ± 1.01 and 4.19 ± 0.89 μmol/min/g tissue, *p* < 0.01; kidney: 5.07 ± 1.92 and 7.73 ± 1.07 μmol/min/g tissue, *p* < 0.01, respectively).

### Tissue Cytochrome C Levels Were Low After Cardiac Arrest

**Figure 2:**
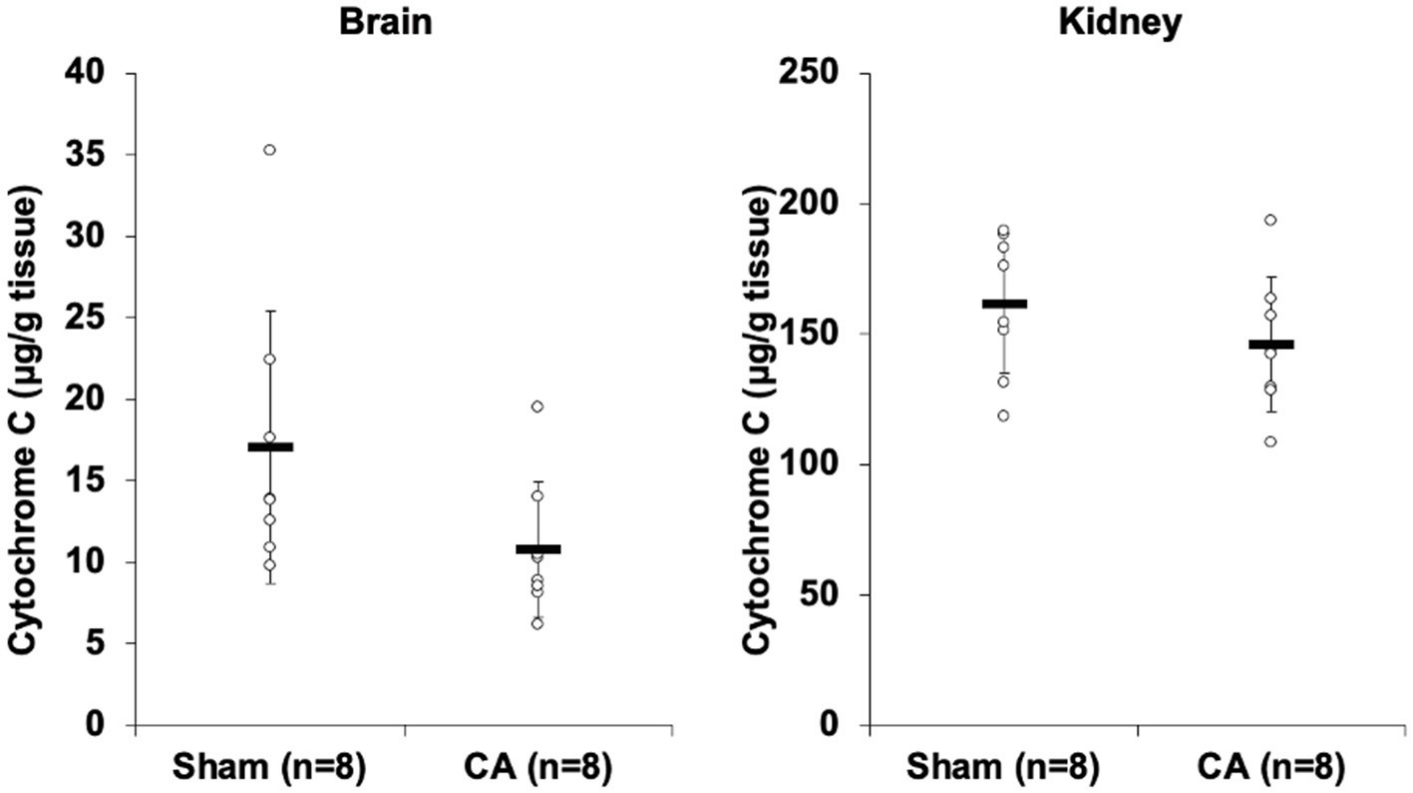
Results of cytochrome C profiling ELISA comparing isolated mitochondria of cardiac arrest rats with that of sham rats. Cytochrome c concentration (μg/g tissue) of isolated mitochondria in the brain and the kidneys. Although there was no statistical difference, cytochrome c levels in the brain and kidney mitochondria of the CA group decreased compared to levels in the sham group (brain, 10.8 ± 4.2 and 17.0 ± 8.4 μg/g tissue, *p* = 0.08; kidney, 146 ± 26 and 162 ± 27 μg/g tissue, *p* = 0.25).

Figure 2 shows the results of cytochrome c profiling ELISA for isolated mitochondria from the brain and kidneys. Although there was no statistical difference, cytochrome c levels in the brain and kidney mitochondria of the CA group decreased compared to levels in the sham group (brain: 10.8 ± 4.2 and 17.0 ± 8.4 μg/g tissue, *p* = 0.08; kidney: 146 ± 26 and 162 ± 27 μg/g tissue, *p* = 0.25).

## Discussion

Our data indicate that 10-min asphyxia CA followed by resuscitation for 30 min decreases mitochondrial respiratory function in the brain and kidneys. These results are in line with our previous findings (12) and other reports on mitochondrial dysfunction in ischemia/reperfusion injury models (13). Although the underlying mechanism for the decreased isolation yield in tissues remains unclear, alterations in cell viability and mitochondrial physiological activity after injury may affect the isolated mitochondrial volume (5). This is supported by our findings that decreased mitochondrial quantity, measured by three independent assays, in the acute-injury rodent model. Considering the potential bias generated by mitochondrial isolation methods and the lack of reliable information on the same, our findings are important for mitochondrial research using acute-injury models.

In addition to being an indicator of mitochondrial quantity in the isolated samples, the protein amount (yield) may represent differences in the mitochondrial content between sham (non-injured) and CA (injured) rats. In other words, the mitochondrial purification method used could affect the sample volume after injury. However, the protein assay was non-specific; therefore, in order to evaluate the decrease in mitochondria-specific quantity in injured tissues, we performed a CS activity assay and cytochrome c ELISA, independent of the number of mitochondrial yields.

CS plays a central role in the mitochondrial oxidative capacity during the first step of the citric acid cycle and is commonly used as a quantitative enzyme marker for the presence of intact mitochondria. Cytochrome c, a key protein involved in cellular respiration, contains an iron porphyrin cofactor. Cytochrome c is primarily involved in the electron transport chain of the mitochondrial inner membrane and is widely believed to be localized solely in the mitochondrial inner membrane space under normal physiological conditions. Because these assays are independent and mitochondria-specific, we used these as independent indicators of mitochondrial quantity in our mitochondria samples.

Collectively, our findings suggest that CA might cause decreased mitochondrial respiration activity and reduced mitochondrial purification in the brain and kidneys. Despite the decrease in mitochondrial quantity in the injured tissues, mitochondrial respiration activity was standardized and adjusted based on the number of yields. The use of standardized values to compare rats with different injury levels is the most widely accepted method worldwide.

Cytochrome c is also known to be extruded into the soluble cytoplasm through pores in the outer mitochondrial membrane during the early phase of apoptosis. In clinical settings, Donnino et al. reported that non-surviving CA patients had higher cytochrome c levels in plasma samples than survivors (14). Accordingly, if we consider that the isolated mitochondria in CA rats could release cytochrome c and mitochondrial fragments into the cytosol and bloodstream, then detection of lower cytochrome c levels might represent a potential selection bias of the samples. This may result in loss of information from damaged mitochondria because of the isolation procedure. Thus, if we could obtain information from damaged/fragmented mitochondria in our mitochondrial functional analysis, the results could be even worse than that observed in the present study, especially as mitochondria require intact inner and outer membranes to complete oxidative phosphorylation.

This study had several limitations. First, our isolation procedure can either increase or decrease the amount of loss in damaged mitochondria from injured tissues. Multiple isolation procedures are still being reported, particularly focusing on the centrifugation speed. The initial centrifugation is key to separating mitochondria from other cytosolic organelles and requires a centrifugation speed ranging from 400*g* to 30,700*g* (15, 16) and 600*g* to 1,000*g* (17, 18) for the brain and kidney samples, respectively. The isolation conditions vary depending on the target tissues, and this probably depends on the investigator's goal. It is difficult to compare our method to those reported previously, as the information regarding their sample quality, which is measured by the yield, CS activity, or cytochrome c level, is not generally reported by the investigators. We selected a balance between low and high centrifugation speeds and used 5,600*g* in our experiment. It is possible that other centrifugation speeds can alter the dignity of sampling bias as compared to those we have shown in this study. However, because centrifugation uses gravity to separate the cytosol organelles, it is plausible that any centrifugation may generate a certain level of bias in the mitochondrial isolation sample. Second, we did not perform mitochondrial DNA measurement, which might have strengthened the results of isolated mitochondrial quantitation, nor did we perform mitochondrial immunostaining of the harvested tissues, which might help understand the morphological and quantitative alterations in the mitochondria after CA. Fan et al. performed histological evaluation of mitochondrial morphology after CA using transmission electron microscopy and found that mitochondria appeared smaller, lost their typical round or tubular morphology, and exhibited an irregular shape after CA (19). Therefore, further histological assessment is of great importance in evaluating the potential sampling bias of mitochondrial isolation methods.

In conclusion, CA decreased mitochondrial respiration activity and the quantity of the mitochondria isolated using our method from the brain and kidneys of rats. Thus, for mitochondrial research, it is important to evaluate the mitochondrial quantity and accordingly adjust the value of mitochondrial function in order to standardize and compare the results obtained under different experimental conditions.

## Data Availability Statement

The raw data supporting the conclusions of this article will be made available by the authors, without undue reservation.

## Ethics Statement

The animal study was reviewed and approved by The Institutional Animal Care and Use Committees of Feinstein Institutes for Medical Research approved this study protocol.

## Author Contributions

KS has full access to all of the data in the study and take responsibility for the integrity of the data and the accuracy of the data analysis and supervised the project. KS and LB designed the conception of the study. KS, TA, and YO performed acquisition of data. TA and YO analyzed data. TA drafted and KS critically edited the manuscript. All authors made interpretations of data and added intellectual content of revisions to the paper and gave full approval of the version to be published.

## Conflict of Interest

KS and LB have a patent right of metabolic measurements in critically ill patients. KS has a grant/research support from Nihon Kohden Corp. LB has a grant/research support from Philips Healthcare, the NIH, Nihon Kohden Corp., Zoll Medical Corp, PCORI, BrainCool, and United Therapeutics and owes patents including 7 issued patents and several pending patents involving the use of medical slurries as human coolant devices to create slurries, reperfusion cocktails, and measurement of respiratory quotient. The remaining authors declare that the research was conducted in the absence of any commercial or financial relationships that could be construed as a potential conflict of interest.

## References

[1] Becker LB Aufderheide TP Graham R. Strategies to improve survival from cardiac arrest. JAMA. (2015) 314:223–4. 10.1001/jama.2015.8454 26132709

[2] Radhakrishnan J Wang S Ayoub IM Kolarova JD Levine RE Gazmuri RJ. Circulating levels of cytochrome c after resuscitation from cardiac arrest: a marker of mitochondrial injury and predictor of survival. Am J Physiol Heart Circ Physiol. (2007) 292:H767–75. 10.1152/ajpheart.00468.2006 17040974 PMC1796625

[3] Yang L Wang J Deng Y Gong C Li Q Chen Q. Melatonin improves neurological outcomes and preserves hippocampal mitochondrial function in a rat model of cardiac arrest. PLoS ONE. (2018) 13:e0207098. 10.1371/journal.pone.0207098 30399193 PMC6219808

[4] Piao L Fang YH Hamanaka RB Mutlu GM Dezfulian C Archer SL. Suppression of superoxide-hydrogen peroxide production at site IQ of mitochondrial complex I attenuates myocardial stunning and improves postcardiac arrest outcomes. Crit Care Med. (2020) 48:e133–40. 10.1097/CCM.0000000000004095 31939812 PMC6964871

[5] Picard M Taivassalo T Ritchie D Wright KJ Thomas MM Romestaing C. Mitochondrial structure and function are disrupted by standard isolation methods. PLoS ONE. (2011) 6:e18317. 10.1371/journal.pone.0018317 21512578 PMC3065478

[6] Kappler L Li J Häring H-U Weigert C Lehmann R Xu G. Purity matters: a workflow for the valid high-resolution lipid profiling of mitochondria from cell culture samples. Sci Rep. (2016) 6:21107. 10.1038/srep21107 26892142 PMC4759577

[7] Hartwig S Feckler C Lehr S Wallbrecht K Wolgast H Muller-Wieland D. A critical comparison between two classical and a kit-based method for mitochondria isolation. Proteomics. (2009) 9:3209–14. 10.1002/pmic.200800344 19415664

[8] Schulz S Lichtmannegger J Schmitt S Leitzinger C Eberhagen C Einer C. A protocol for the parallel isolation of intact mitochondria from rat liver, kidney, heart, and brain. Methods Mol Biol. (2015) 1295:75–86. 10.1007/978-1-4939-2550-6_7 25820715

[9] Shinozaki K Becker LB Saeki K Kim J Yin T Da T. Dissociated oxygen consumption and carbon dioxide production in the post-cardiac arrest rat: a novel metabolic phenotype. J Am Heart Assoc. (2018) 7:e007721. 10.1161/JAHA.117.007721 29959138 PMC6064898

[10] Scholte HR Yu Y Ross JD Oosterkamp II Boonman AM Busch HF. Rapid isolation of muscle and heart mitochondria, the lability of oxidative phosphorylation and attempts to stabilize the process in vitro by taurine, carnitine and other compounds. Mol Cell Biochem. (1997) 174:61–6. 10.1007/978-1-4615-6111-8_9 9309666

[11] Krahenbuhl S Chang M Brass EP Hoppel CL. Decreased activities of ubiquinol:ferricytochrome c oxidoreductase (complex III) and ferrocytochrome c:oxygen oxidoreductase (complex IV) in liver mitochondria from rats with hydroxycobalamin[c-lactam]-induced methylmalonic aciduria. J Biol Chem. (1991) 266:20998–1003. 10.1016/S0021-9258(18)54810-9 1657942

[12] Kim J Villarroel JP Zhang W Yin T Shinozaki K Hong A. The responses of tissues from the brain, heart, kidney, and liver to resuscitation following prolonged cardiac arrest by examining mitochondrial respiration in rats. Oxid Med Cell Longev. (2016) 2016:7463407. 10.1155/2016/7463407 26770657 PMC4685127

[13] Wu J Li Y Yang P Huang Y Lu S Xu F. Novel role of carbon monoxide in improving neurological outcome after cardiac arrest in aged rats: involvement of inducing mitochondrial autophagy. J Am Heart Assoc. (2019) 8:e011851. 10.1161/JAHA.118.011851 31030597 PMC6512094

[14] Donnino MW Liu X Andersen LW Rittenberger JC Abella BS Gaieski DF. National Post Arrest Research Consortium, Characterization of mitochondrial injury after cardiac arrest (COMICA). Resuscitation. (2017) 113:56–62. 10.1016/j.resuscitation.2016.12.029 28126408 PMC5497747

[15] Racay P Chomova M Tatarkova Z Kaplan P Hatok J Dobrota D. Ischemia-induced mitochondrial apoptosis is significantly attenuated by ischemic preconditioning. Cell Mol Neurobiol. (2009) 29:901–8. 10.1007/s10571-009-9373-7 19283470 PMC11505820

[16] Abe T Takagi N Nakano M Takeo S. The effects of monobromobimane on neuronal cell death in the hippocampus after transient global cerebral ischemia in rats. Neurosci Lett. (2004) 357:227–31. 10.1016/j.neulet.2003.12.088 15003291

[17] Beiral HJ Rodrigues-Ferreira C Fernandes AM Gonsalez SR Mortari NC Takiya CM. The impact of stem cells on electron fluxes, proton translocation, and ATP synthesis in kidney mitochondria after ischemia/reperfusion. Cell Transplant. (2014) 23:207–20. 10.3727/096368912X659862 23211430

[18] Muthuraman A Sood S Ramesh M Puri KD Peters A Chauhan A. Therapeutic potential of 7,8-dimethoxycoumarin on cisplatin- and ischemia/reperfusion injury-induced acute renal failure in rats. Naunyn Schmiedebergs Arch Pharmacol. (2012) 385:739–48. 10.1007/s00210-012-0751-1 22526471

[19] Fan J Cai S Zhong H Cao L Hui K Xu M. Therapeutic hypothermia attenuates global cerebral reperfusion-induced mitochondrial damage by suppressing dynamin-related protein 1 activation and mitochondria-mediated apoptosis in a cardiac arrest rat model. Neurosci Lett. (2017) 647:45–52. 10.1016/j.neulet.2017.02.065 28242326

